# Mesenchymal stromal cells expressing a dominant‐negative high mobility group A1 transgene exhibit improved function during sepsis

**DOI:** 10.1002/JLB.4A0720-424R

**Published:** 2021-01-13

**Authors:** Min‐Young Kwon, Sailaja Ghanta, Julie Ng, Ana P. Castano, Junwen Han, Bonna Ith, James A. Lederer, Souheil El‐Chemaly, Su Wol Chung, Xiaoli Liu, Mark A. Perrella

**Affiliations:** ^1^ Division of Pulmonary and Critical Care Medicine Department of Medicine Brigham and Women's Hospital and Harvard Medical School Boston Massachusetts USA; ^2^ Department of Pediatric Newborn Medicine Brigham and Women's Hospital and Harvard Medical School Boston Massachusetts USA; ^3^ Department of Surgery Brigham and Women's Hospital and Harvard Medical School Boston Massachusetts USA; ^4^ Department of Biological Sciences University of Ulsan Ulsan South Korea

**Keywords:** architectural transcription factor, bacterial infection, inflammation, mesenchymal stromal cells, neutrophil function

## Abstract

High mobility group (HMG)A proteins are nonhistone chromatin proteins that bind to the minor groove of DNA, interact with transcriptional machinery, and facilitate DNA‐directed nuclear processes. HMGA1 has been shown to regulate genes involved with systemic inflammatory processes. We hypothesized that HMGA1 is important in the function of mesenchymal stromal cells (MSCs), which are known to modulate inflammatory responses due to sepsis. To study this process, we harvested MSCs from transgenic (Tg) mice expressing a dominant‐negative (dn) form of HMGA1 in mesenchymal cells. MSCs harvested from Tg mice contained the dnHMGA1 transgene, and transgene expression did not change endogenous HMGA1 levels. Immunophenotyping of the cells, along with trilineage differentiation revealed no striking differences between Tg and wild‐type (WT) MSCs. However, Tg MSCs growth was decreased compared with WT MSCs, although Tg MSCs were more resistant to oxidative stress‐induced death and expressed less IL‐6. Tg MSCs administered after the onset of *Escherichia coli*‐induced sepsis maintained their ability to improve survival when given in a single dose, in contrast with WT MSCs. This survival benefit of Tg MSCs was associated with less tissue cell death, and also a reduction in tissue neutrophil infiltration and expression of neutrophil chemokines. Finally, Tg MSCs promoted bacterial clearance and enhanced neutrophil phagocytosis, in part through their increased expression of stromal cell‐derived factor‐1 compared with WT MSCs. Taken together, these data demonstrate that expression of dnHMGA1 in MSCs provides a functional advantage of the cells when administered during bacterial sepsis.

## Graphical Abstract

Administration of adipose‐derived MSCs expressing a dominant‐negative HMGA1 transgene results in less tissue injury and inflammation during a systemic infection, while improving neutrophil phagocytosis.

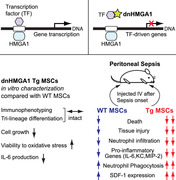

**Abbreviations**
CLP: cecal ligation and puncture
Cox: cyclooxygenase
SDF‐1: stromal cell‐derived factor‐1
ESCs: embryonic stem cells
dn: dominant‐negative
HMG: high mobility group
MSCs: mesenchymal stromal cells
PIS: proinflammatory stimuli
qRT‐PCR: quantitative real time polymerase chain reaction
RT: reverse transcriptase
Tg: transgenic
WT: wild‐type

## 1 INTRODUCTION

High mobility group (HMG)A proteins are architectural factors that modify the structure of chromatin in a dynamic fashion.^1^ These nonhistone chromatin proteins facilitate DNA‐directed nuclear processes, including transcription, replication, and repair.^2^, ^3^ The HMGA family contains a functional motif of AT‐hooks, which characterize the family, and these hooks bind to AT‐rich regions in the minor groove of DNA.^4^, ^5^ Binding of HMGA proteins to DNA promotes conformational changes in the DNA that can either facilitate or prevent the assembly of enhanceosome complexes, resulting in changes in gene transcription.^6^ Through these interactions with DNA and other proteins, HMG proteins influence biologic processes such as cell growth, proliferation, differentiation, and death.

The expression of HMG proteins, such as HMGA1, is higher in undifferentiated and embryonic cells, including stem cells and cancer cells, and is linked to transcriptional networks that drive pluripotency and cellular proliferation.^1^, ^7^, ^8^, ^9^, ^10^ During cellular differentiation, and in adult terminally differentiated cells, the expression of HMGA1 is decreased.^10^, ^11^ Although HMGA1 is expressed at a lower level in adult, differentiated tissues, the expression of HMGA1 can increase under disease conditions. We have previously shown that vascular smooth muscle cells have low baseline levels of HMGA1. However, expression can be induced by exposure to bacterial endotoxin, the proinflammatory cytokine IL‐1β, and to the proliferative stimulus of serum, both in vitro and in vivo.^12^, ^13^

To better understand the importance of HMGA1 in mesenchymal cells, we generated a transgenic (Tg) mouse overexpressing a dominant‐negative (dn) form of HMGA1 targeted to mesenchymal cells of vascular smooth muscle origin.^14^ This dn transgene is not capable of binding to DNA; however, it interacts with transcription factors. The dnHMGA1 Tg mice were less hypotensive to endotoxemia and had lower mortality in bacterial sepsis. In addition, the Tg mice had reduced tissue infiltration of inflammatory cells during experimental sepsis.^14^ As mesenchymal stromal cells (MSCs) are another mesenchymal cell with a significant influence on the inflammatory response during sepsis,^15^, ^16^, ^17^ we expanded our appraisal of HMGA1 from vascular smooth muscle cells to MSCs. We decided to study adipose‐derived MSCs of the Tg mice, a cell population located in the stromal vascular fraction of adipocyte tissue. After determining that the dnHMGA1 transgene was expressed in these MSCs, the focus of the present study was to investigate whether disruption of the HMGA1 pathway would change the MSC phenotype in vitro, and have a functional impact on the cells when they were administered during bacterial sepsis in vivo. Our hypothesis was that overexpression of the dnHMGA1 transgene in adipose‐derived MSCs would improve their ability to mediate the inflammatory response, reduce tissue injury, and improve outcome when administered after the onset of bacterial sepsis.

## 2 MATERIALS AND METHODS

### 2.1 Isolation, characterization, and differentiation assays of murine MSCs

MSCs were harvested from inguinal fat pads of wild‐type (WT) and dnHMGA1 Tg (specifically Tg1^14^) littermate mice. The Tg mice were generated using a dnHMGA1 construct containing 4 proline to alanine substitutions introduced at amino acids 57, 61, 83, and 87 located in the second and third DNA‐binding domains (AT‐hooks), as described previously,^18^ which prevents the dn construct from binding to DNA.^14^ The inguinal fat pads were washed and minced, digested enzymatically with 0.1% collagenase I and 0.25% collagenase II, and the cells were filtered via a 70 μm cell strainer and centrifuged at 300 *g* for 10 min at room temperature as described.^19^ The supernatant was removed, and the cells were plated for expansion using MesenCult Proliferation Kit (StemCell Technologies, Vancouver, BC).

The cells were characterized using a BD fluorescent‐activated cell sorting (FACS) Canto II (BD Biosciences, Billerica, MA), and analyzed using FlowJo software. The antibodies used for phenotyping of cells are listed in Supplemental Table 1. Multilineage differentiation capacity of MSCs into osteoblasts, adipocytes, and chondrocytes was also performed as described.^17^, ^19^, ^20^

Additionally, the cells were assessed for growth potential. WT and Tg MSCs were placed in low serum medium (1% FBS) overnight, and then changed to full growth medium using the MesenCult Proliferation Kit (StemCell Technologies, Vancouver, BC). The number of MSCs in each of the groups was quantified at baseline in low serum medium, and then daily over the next 5 days in full growth medium using a hemocytometer.

### 2.2 Assessment of adipose‐derived MSCs by qRT‐PCR

Total RNA was extracted from WT and dnHMGA1 Tg MSCs by TRIzol^®^ reagent (Thermo Fisher Scientific, Waltham, MA). qRT‐PCR with SYBR Green Master Mix (Bio‐Rad Laboratories, Hercules, CA) was performed using the StepOnePlus Real‐Time PCR System (Applied Biosystems, Foster City, CA) as described.^14^, ^21^ The samples were treated with DNase I to degrade any genomic DNA contamination, and cDNA synthesized by iScript™ Reverse Transcription Supermix (Bio‐Rad Laboratories). qRT‐PCR was performed for mouse HMGA1 using primers forward 5′‐GCTGGTCGGAGTCAGAAAG‐3′ and reverse 5′‐GGTGACTTTCCGGGTCTTGG‐3′; mouse cyclooxygenase‐2 (Cox‐2) using primers forward 5′‐GCCTACTACAAGTGTTTCTTTTTGCA‐3′ and reverse 5′‐CATTTTGTTTGATTGTTCACACCAT‐3′; mouse IL‐6 using primers forward 5′‐ACAAGTCGGAGGCTTAATTACACAT‐3′ and reverse 5′‐TTGCCATTGCACAACTCTTTTC‐3′; mouse MIP‐2 using primers forward 5′‐CCACCAACCACCAGGCTACAGGGGC‐3′ and reverse 5′‐AGGCTCCTCCTTTCCAGGTCAGTTAGC‐3′, mouse KC using primers forward 5′‐ACCCGCTCGCTTCTCTGT‐3′ and reverse 5′‐AAGGGAGCTTCAGGGTCAAG‐3′, and mouse stromal cell‐derived factor‐1 (SDF‐1) using primers forward 5′‐GAGCCAACGTCAAGCATCTG ‐3′ and reverse 5′‐CGGGTCAATGCACACTTGTC‐3′. qRT‐PCR of β‐actin was employed for normalization of gene expression by the comparative Ct method, using primers of mouse β‐actin forward 5′‐ACCAACTGGGACGATATGGAGAAGA‐3′ and reverse 5′‐TACGACCAGAGGCATACAGGGACAA‐3′.

### 2.3 Chemokine/cytokine assay

For IL‐6 analysis of cell and tissue extracts, samples were assessed using the multiplex assay technology by Luminex as described previously.^22^, ^23^ The levels of IL‐6 were determined by standard curve analysis. The plate was read on a Luminex 200TM instrument (Luminex, Austin, TX). Data acquisition and analysis were conducted using StarStation software v2.3 (Applied Cytometry Systems, Dinnington, UK). In addition, cell culture supernatants were collected from MSCs, and SDF‐1 was measured using a commercially available ELISA kit (R&D, Minneapolis, MN) according to the manufacturer's instructions.^24^

### 2.4 Silencing of SDF‐1

For the silencing of SDF‐1, target sequence CATCAGTGACGGTAAACCAGTC(consortium number TRCN0000195944), or a scrambled (SCR) control sequence (SHC016) were obtained from Sigma‐Aldrich (St. Louis, MO, USA). Lentiviral particles were generated using a commercially available packaging mix (Sigma‐Aldrich; SHP001) in human embryonic kidney 293 T cells, according to the manufacturer's instructions. The WT and Tg MSCs were infected with the lentiviral particles, and stably selected by use of puromycin (10 μg/ml) as described.^20^ The effectiveness of SDF‐1 silencing was assessed at the protein level by ELISA (see above).

### 2.5 Reagents

Murine recombinant IL‐1β, IFN‐γ, and TNF‐α were purchased from PeproTech, Inc (Rocky Hill, NJ, USA). The cytokines were administered together (each at a dose of 10 ng/ml) to produce a proinflammatory stimulus to MSCs. *Escherichia coli* LPS (serotype O26:B6) was purchased from Sigma–Aldrich, and administered to MSCs at a dose of 100 ng/ml.

### 2.6 Models of peritoneal sepsis in mice

Fibrin clots of *E. coli* bacteria (strain MMB1287) were generated using an analogous number of bacteria as described previously.^14^ Under ketamine/xylazine anesthesia and sterile conditions, the fibrin clot was placed within the peritoneal cavity of C57BL/6 male WT and Tg mice via a 1.5 cm abdominal incision that was closed in layers with 6–0 surgical sutures (Harvard Apparatus, Holliston, MA). Mice initially received WT or dnHMGA1 Tg MSCs (2.5 × 10^5^ cells/200 μl PBS), a control mesenchymal cell (fibroblasts in 200 μl PBS), or vehicle (PBS 200 μl), via intravenous administration at 6 and 24 h after fibrin clot to assess survival. Mice subsequently received MSCs or vehicle (as above) in a single dose at 6 h after fibrin clot for survival and functional studies. Depending on the experiment, the mice were either sacrificed at 24 h, or they were monitored over a 7‐day period to determine survival.

Polymicrobial sepsis was induced by cecal ligation and puncture (CLP), as described.^17^, ^25^, ^26^ Briefly, C57BL/6 male mice 7–9 weeks of age were anesthetized, the peritoneum was opened, and two‐thirds of the cecum was ligated and punctured with 2 21‐gauge holes. In sham experiments, the same procedure was performed; however, CLP was not performed. The mice received either WT or dnHMGA1 Tg MSCs (2.5 × 10^5^ cells/200 μl PBS) or vehicle (PBS 200 μl) via intravenous administration at 6 h after CLP. In the CLP experiments, the mice also received an antibiotic,^27^ Imipenem (Sigma; 25 mg/kg subcutaneously), at the time of MSC administration. The mice were sacrificed at 24 h after CLP or sham surgery.

### 2.7 Histology and inflammatory cell infiltration

Mice were sacrificed 24 h following fibrin clot placement or CLP, and the spleens and lungs were harvested for histologic evaluation. The tissues were harvested as described^14^ and immunostained with a Ly6G (BioLegend, Dedham, MA) antibody for assessment of neutrophil infiltration, and scored by an investigator who was blinded to the group. Positively stained cells were evaluated per 20× objective, and numerous random fields were assessed per tissue section using Image J software. Cells from the peritoneal fluid were stained with an antibody targeting Ly6G‐APC (#127613; BioLegend), to identify neutrophils. The cells were then assessed by flow cytometry using a BD FACS Canto II, and analyzed by FlowJo software.

### 2.8 Assessment of cell death in tissue

Terminal deoxynucleotidetransferase‐mediated dUTP nick end‐labeling (TUNEL assay) Clontech (Mountain View, CA, USA) was used to detect apoptotic cell death in tissue sections of lungs and spleens from mice in each group per the manufacturer's protocol. Quantification was performed as described for Ly6G staining.

### 2.9 Bacterial CFUs of peritoneal lavage

Mice underwent fibrin clot or sham surgery. The mice were then sacrificed 24 h after surgery, and peritoneal lavages were performed.^17^ Serial dilutions of the peritoneal fluid were made, plated on LB agar plates, and then incubated overnight. CFUs were counted and calculated as described.^17^

### 2.10 Isolation of murine neutrophils

For the isolation of neutrophils, mice were given an intraperitoneal injection of Bio‐Gel P100 polyacrylamide beads (2% solution; Bio‐Rad Laboratories) as described.^17^ After 24 h, the mice were anesthetized and 10 ml of sterile PBS was used to lavage the peritoneal cavity, and cells were washed and placed in RPMI 1640 medium with 0.3% BSA and 10 mM 4‐(2‐hydroxyethyl)‐1‐piperazineethanesulfonic acid.

### 2.11 Phagocytosis assay

MSCs were added at a ratio of 1 MSC to 5 neutrophils. Isolated neutrophils were activated with 10 ng/ml of G‐CSF (mouse) for 2 h. Green fluorescent protein (GFP)‐labeled *E. coli* (strain MMB1287) were then added at 10 multiplicity of infection per neutrophil. Bacterial phagocytosis was measured by flow cytometry as described.^17^, ^20^

### 2.12 Study approval

Studies using mice were carried out in accordance with the Public Health Service policy on the humane care and use of laboratory animals, and approved by the Institutional Animal Care and Use Committee (IACUC) of Brigham and Women's Hospital.

### 2.13 Statistics

For comparisons between 2 groups, we used Student's unpaired *t*‐test. For analysis of more than 2 groups, one‐way ANOVA was used. The assessment of cell growth between groups, over a period of 5 days, was analyzed by two‐way ANOVA. When data were not normally distributed, nonparametric analyses were performed using Mann–Whitney *U* or Kruskal–Wallis testing, respectively. Comparisons of mortality were made by analyzing Kaplan–Meier survival curves, and then log‐rank test to assess for differences in survival. Statistical significance was accepted at *P *< 0.05.

## 3 RESULTS

### 3.1 Characterization of dnHMGA1 Tg MSCs

MSCs were harvested from adipose tissue of WT and dnHMGA1 Tg mice. The dnHMGA1 transgene was present in the Tg cells, but not WT cells (Fig. 1A). Expression of the transgene did not alter endogenous mRNA levels of HMGA1, which was not different between WT MSCs and dnHMGA1 Tg MSCs (Fig. 1B). However, Cox‐2 mRNA (known to be regulated by HMGA1^28^) was suppressed in dnHMGA1 Tg MSCs, and induction of Cox‐2 by LPS was abolished (Fig. 1C). These data support that the dnHMGA1 transgene is functional in Tg MSCs. The phenotype of the MSCs was also assessed by flow cytometry. WT and dnHMGA1 Tg MSCs showed comparable expression of mesenchymal markers CD105, CD73, CD90.2, CD29, CD44, and Stro1 (Fig. 1D and E). Moreover, in both lines of MSCs, Sca1 was highly expressed, whereas a very low percentage of cells expressed makers of hematopoietic origin (CD45 and c‐kit) or major histocompatibility complex II. We next investigated the trilineage differentiation of WT and dnHMGA1 Tg MSCs. Figure 1F demonstrates that both lines of MSCs differentiated into osteoblasts, adipocytes, and chondrocytes. These data suggest that overexpression of the dnHMGA1 construct does not alter the expression of mesenchymal markers or the differentiation potential of MSCs.

FIGURE 1
**Characterization of adipocyte‐derived MSCs harvested from dominant‐negative (DN) HMGA1 transgenic (Tg) mice**. (**A**) Total RNA was extracted from wild‐type (WT) and dnHMGA1 Tg MSCs, and reverse transcriptase PCR was performed using 5′ and 3′ primers designed according to the sequences of the dnHMGA1 cDNA construct and the HA‐tag designed to generate the mice.^14^ PCR for beta (β)‐actin was used as control for gene expression. (**B**) WT and dnHMGA1 Tg MSCs were also assessed by quantitative real time PCR (qRT‐PCR) for the level of endogenous HMGA1. qRT‐PCR of β‐actin was employed for normalization of gene expression. Gene expression is presented as fold‐increase compared with WT MSCs. Data are presented as mean ± sem, *n* = 3. Analysis by 2‐tailed unpaired *t*‐test; NS, not significant. (**C**) qRT‐PCR was used to assess Cox‐2 mRNA levels at 24 h after LPS (100 ng/ml) or vehicle, whereas β‐actin was employed for normalization of gene expression. Gene expression is presented as fold‐increase compared with WT MSCs. Data are presented as mean ± sem, from 2 independent experiments. Analysis by one‐way ANOVA (*P *< 0.0001) with significant comparisons * versus WT MSCs no (−) LPS, and † versus WT MSCs + LPS. (**D** and **E**) Flow cytometry characterization was performed of WT and dnHMGA1 Tg MSCs. After expansion and depletion of the hematopoietic lineage cells, putative MSCs were assessed for mesenchymal markers (CD105, CD73, CD90.2, CD29, CD44, Stro1), Sca‐1, c‐kit, hematopoietic origin marker CD45, and MHC II. Representative histograms are shown for WT MSCs (**D**) and dnHMGA1 Tg MSCs (**E**), with staining for isotype control antibodies (white) and target antibodies (gray). (**F**) Representative light microscope images of putative MSCs (WT and dnHMGA1 Tg) grown in specialized media to promote differentiation of osteoblasts (left panel, Alizarin Red S stain), adipocytes (middle panel, Oil‐red‐O stain), and chondrocytes (right panel, toluidine blue stain)

**Figure:**
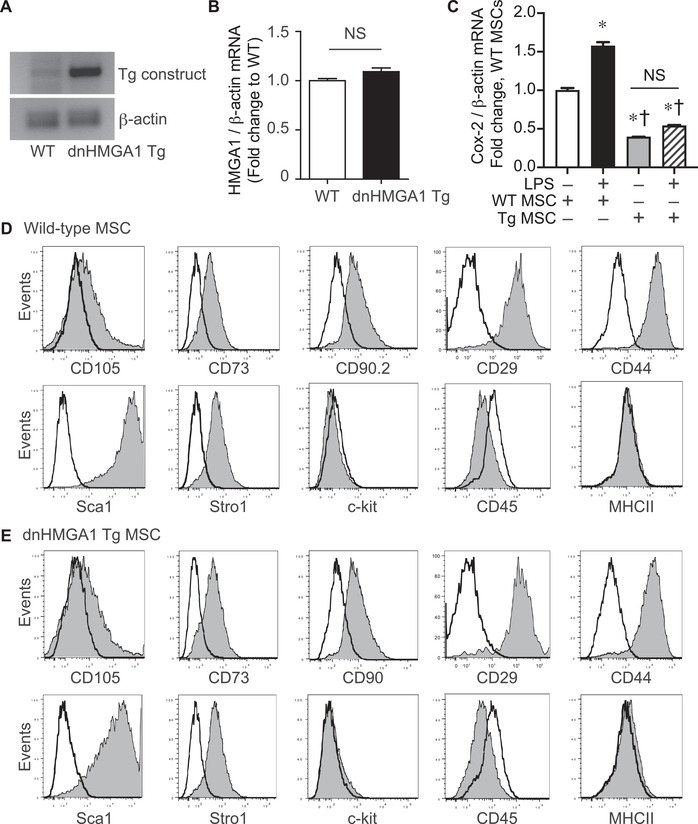

**Figure:**
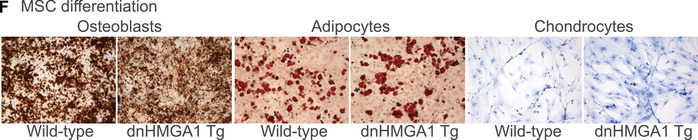

### 3.2 MSC cell growth and response to oxidative stress/inflammatory stimuli

The number of MSCs in the WT and Tg groups was counted at baseline in low serum medium, and then over the next 5 days in full growth medium. Cell growth, as depicted by cell number in each group of MSCs, demonstrated that Tg MSCs have significantly fewer cells on days 3, 4, and 5 compared with WT MSCs (Fig. 2A).

FIGURE 2
**Transgenic (Tg) expression of dnHMGA1 suppressed the growth response, and resulted in less cell death and IL‐6 production in the presence of oxidative stress and inflammatory stimuli**. (**A**) Wild‐type (WT) and dnHMGA1 Tg MSCs were assessed for cell growth. The number of MSCs in each of the groups was quantitated at baseline in low serum medium (0 day), and then daily over the next 5 days in full growth medium. Data are represented as cell number (×10^4^), in 3 independent experiments. Analysis by two‐way ANOVA (*P *= 0.0095) with significant comparison, * versus WT MSCs using Bonferroni's multiple comparisons test. (**B**) WT and dnHMGA1 Tg MSCs were plated, and 24 h later the cells were treated with H_2_O_2_ (125 μmol/l) for 24 h. Control groups were left untreated. 3‐(4,5‐Dimethylthiazol‐2‐yl)2,5‐diphenyl‐tetrazolium bromide (MTT) assay for living cells was performed, and quantified by reading absorbance at 590 nm. Data are shown as MTT absorbance compared with the absorbance of the untreated WT MSCs and expressed as a percentage. Data are expressed as mean ± sem (*n* = 3 in control and *n* = 7 in H_2_O_2_ groups). Analysis by one‐way ANOVA (*P *< 0.001) with significant comparison * versus no (–) H_2_O_2_ groups, † versus WT MSCs + H_2_O_2_ using Tukey's multiple comparisons test. (**C**) WT and dnHMGA1 Tg MSCs were stimulated with proinflammatory stimuli (PIS) mIL‐1β + mIFN‐γ + mTNF‐α for 12 h. Luminex analysis of cell lysates was then performed for IL‐6. Bar graphs are depicted as mean ± sem (pg/ml) of 3 independent experiments. Analysis by one‐way ANOVA (*P *< 0.0001) with significant comparisons * versus both no (–) PIS groups, and † versus WT MSCs + PIS using Tukey multiple comparisons analysis

**Figure:**
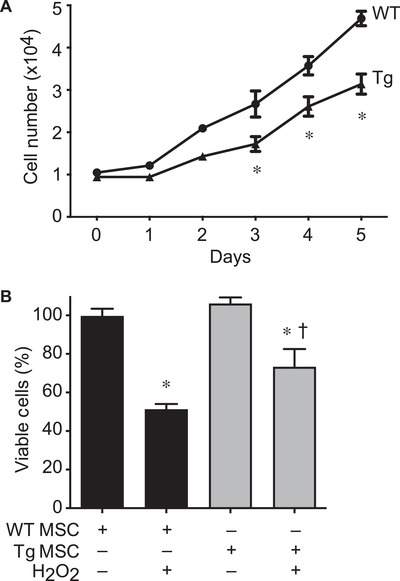

**Figure:**
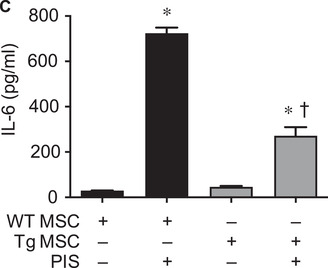

We next exposed the cells to oxidative stress, an important component of the pathophysiologic injury response during sepsis, using hydrogen peroxide (H_2_O_2_).^19^, ^29^ WT MSCs exposed to 125 μM H_2_O_2_ resulted in a significant decrease in viability, with 50.0 ± 2.6% of cells alive at 24 h (Fig. 2B). In contrast, Tg MSCs had more cell survival when exposed to H_2_O_2_, with 74.3 ± 8.2% viable cells. Finally, we assessed the production of IL‐6, an inflammatory mediator important in the pathobiology of sepsis with higher levels associated with worse clinical outcome.^30^, ^31^ Tg MSCs produced less IL‐6 protein after exposure to the proinflammatory stimuli (PIS: mIL‐1β + mIFN‐γ + mTNF‐α) than WT MSCs (Fig. 2C). Taken together, these data demonstrated that even though the growth response of dnHMGA1 Tg MSCs is reduced compared with WT MSCs, the dnHMGA1 TG cells are more resistant to oxidative stress‐induced death and they expressed less IL‐6 than WT cells.

### 3.3 Administration of dnHMGA1 Tg MSCs after the onset of *E. coli*‐induced sepsis exhibits improved cell efficacy with increased survival and decreased tissue injury

Mice were injected with WT and Tg MSCs (2.5 × 10^5^, in 200 μl of PBS) at 6 and 24 h after the onset of *E. coli*‐induced sepsis using the peritoneal fibrin clot model. Injection of MSCs was compared with either a control mesenchymal cell (fibroblasts, 2.5 × 10^5^ in 200 μl PBS) or PBS vehicle (200 μl). When using 2 injections of cells, mice receiving WT or Tg MSCs both showed a significant improvement in survival compared with mice receiving fibroblasts or PBS (Fig. 3A). However, when mice received only 1 injection of MSCs (fewer total cells) at 6 h after the onset of sepsis, the WT cells lost their beneficial response, whereas the Tg cells maintained their efficacy with improved survival of mice (Fig. 3B). In addition, assessment of organs, both inside the peritoneum (spleen) and outside the peritoneum (lung), 24 h after the onset of sepsis showed a decrease in cell death (TUNEL positive cells) in mice receiving WT cells compared with PBS; however, there was a further decrease in cell death in mice receiving Tg MSCs compared with mice receiving WT MSCs (Fig. 3C).

FIGURE 3
**Administration of dnHMGA1 Tg MSCs improved efficacy of the cells resulting in increased survival and decreased tissue cell death in *E. coli*‐induced sepsis**. (**A**) C57BL/6 male mice were randomly separated into 4 groups: PBS, dashed gray line, *n* = 9; fibroblasts, solid gray line, *n* = 9; WT MSCs, dashed black line, *n* = 16; and dnHMGA1 Tg MSCs, solid black line, *n* = 16. All mice were subjected to fibrin clot (*E. coli*) sepsis, and 6 and 24 h later the mice received 2.5 × 10^5^ cells/200 μl PBS or 200 μl PBS by tail vein injection. Survival of mice was monitored for 7 days and data are presented as a Kaplan–Meier survival curve, and analyzed by Log‐rank test (*P *= 0.0142). ∗ MSCs versus PBS; † MSCs versus fibro. (**B**) C57BL/6 mice were then randomly separated into 2 groups: WT MSCs, dashed black line, *n* = 16; and dnHMGA1 Tg MSCs, solid black line, *n* = 16. All mice were subjected to fibrin clot (*E. coli*) sepsis, and after 6 h the mice were treated with 2.5 × 10^5^ cells by tail vein injection. Survival of mice and data analysis as described in (**A**). ∗ dnHMGA1 Tg MSCs versus WT MSCs, *P *= 0.0407. (**C**) C57BL/6 mice received sham surgery (– *E. coli*) or fibrin clot (+ *E. coli*) sepsis. After 6 h, the septic mice received PBS, WT MSCs, or dnHMGA1 Tg MSCs. After 24 h from surgery the lungs and spleens were harvested, and TUNEL staining was performed and assessed as fold change in positive cells compared with – *E. coli*. Data are expressed as mean ± sem (*n* = 12–20 images per group). Analysis by one‐way ANOVA (*P *< 0.0001) with significant comparison * versus sham, † versus PBS, § versus WT MSCs using Tukey's multiple comparisons test

**Figure:**
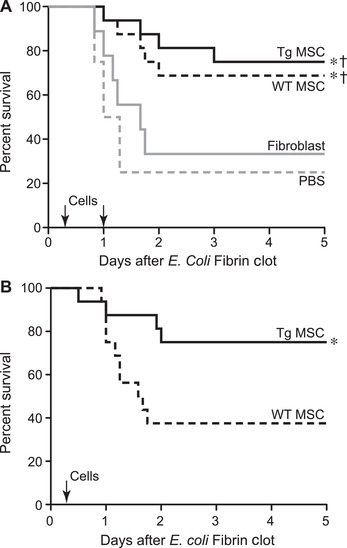

**Figure:**
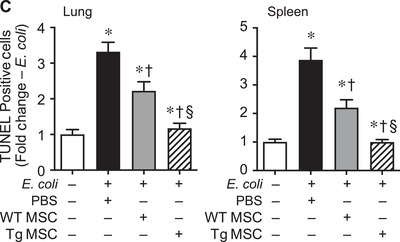

### 3.4 No alteration in peritoneal fluid, but decreased tissue inflammatory response in septic mice receiving dnHMGA1 MSCs

**FIGURE 4:**
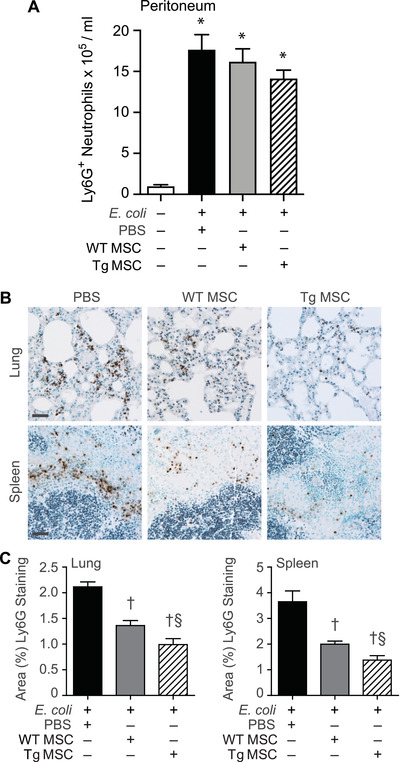
**Administration of dnHMGA1 Tg MSCs during *E. coli*‐induced sepsis decreased Ly6G^+^ neutrophil infiltration into lung and spleen tissue, but not in peritoneal fluid**. C57BL/6 mice underwent fibrin clot (*E. coli*) surgery. Six hours after surgery the mice received PBS, WT MSCs (2.5 × 10^5^), or dnHMA1 Tg MSCs (2.5 × 10^5^). At 24 h after surgery, the peritoneum was lavaged and tissues were harvested. (**A**) The peritoneal fluid was assessed by flow cytometry for Ly6G positive (+) neutrophils. Data are presented as mean ± sem, *n* = 6 in sham and *n* = 11–12 in sepsis groups. Analysis by one‐way ANOVA (*P *< 0.0001) with significant comparison * versus sham. (**B**) Immunostaining was performed with Ly6G antibody (brown) to assess neutrophils in tissues, with representative light microscopy images of lung (upper panel) and spleen (lower panel) of each group. The scale bars represent 25 μm. (**C**) The area of Ly6G positively stained cells from images of lung (left panel) and spleen (right panel) tissue from mice receiving PBS (black bars), WT MSCs (gray bars), and dnHMGA1 Tg MSCs (striped bars) are shown. Area (%) of Ly6G staining was calculated per 20× objective, and random fields were assessed per tissue section. Data are represented as mean ± sem, *n* = 16 in lung and *n* = 11–19 in spleen groups. Analysis by one‐way ANOVA (*P *< 0.0001) with significant comparison † versus PBS, § versus WT MSCs using Holm–Sidak's multiple comparisons test

**FIGURE 5:**
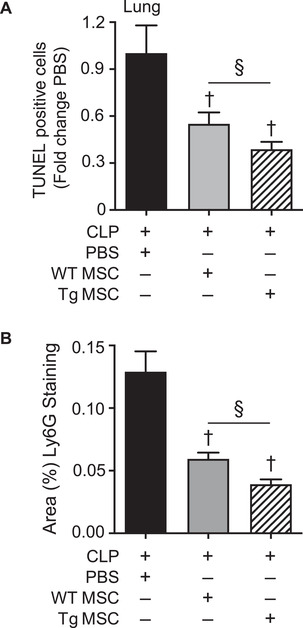
**Administration of dnHMGA1 Tg MSCs during CLP‐induced sepsis plus antibiotics led to decreased cell death and Ly6G^+^ neutrophil infiltration into lung tissue**. C57BL/6 mice underwent CLP‐induced sepsis, and 6 h after surgery received PBS, WT MSCs (2.5 × 10^5^), or dnHMA1 Tg MSCs (2.5 × 10^5^). In addition, in each of the groups, the mice received Imipenem (25 mg/kg subcutaneously) at the time of MSC administration. After 24 h from surgery, the lungs were harvested and stained for TUNEL (**A**) and Ly6G (**B**). TUNEL staining was assessed as fold change in positive cells compared with PBS, and area (%) of Ly6G staining was calculated per 20× objective. The tissue was either scanned for TUNEL staining, or random fields were assessed per tissue section for Ly6G staining. Data are represented as mean ± sem, *n* = 4–5 lungs with assessment of at least 20 images per group. Analysis by one‐way ANOVA (*P *< 0.0001) with significant comparison † versus PBS, § WT MSCs versus dnHMGA1 Tg MSCs using Fisher's LSD test

Assessment of neutrophils was performed in the peritoneal fluid and in the lungs and spleens of mice at 24 h after *E. coli*‐induced fibrin clot, after receiving a single dose of WT MSCs, Tg MSCs, or vehicle (PBS). Flow cytometry of peritoneal fluid revealed increased Ly6G^+^ neutrophils compared with sham (Fig. 4A); however, no differences in neutrophil counts between mice receiving MSCs (WT or Tg) or vehicle (PBS). Representative images of Ly6G^+^ cells by immunostaining is shown in Figure 4B. Quantification of cell infiltration (Fig. 4C) revealed decreased neutrophils in the lungs of mice receiving WT MSCs compared with PBS, and a further decrease in septic mice receiving Tg MSCs. A similar response was found in the spleen, with a decrease in neutrophils in mice receiving Tg MSCs compared with WT MSCs. In the CLP‐model of polymicrobial sepsis plus the antibiotic Imipenem, a parallel tissue pattern was seen as Tg MSCs were able to further decrease TUNEL‐positive cells and the infiltration of Ly6G^+^ neutrophils into lungs compared with mice receiving WT MSCs (Fig. 5A and B).

With the reduced infiltration of neutrophils in the tissue of septic mice receiving Tg MSCs, we next assessed tissue expression of the inflammatory cytokine IL‐6 and neutrophil chemokines KC and MIP‐2. In these studies, we focused on the lung, an organ with inflammation and injury that contributes significantly to the pathobiology of sepsis. Compared with the marked increase in IL‐6 mRNA in the lungs of septic mice receiving PBS, the level of IL‐6 was significantly decreased in mice receiving Tg MSCs, and this level was also decreased compared with mice receiving WT MSCs (Fig. 6A). Similar to the decreased infiltration of Ly6G^+^ cells, the expression of MIP‐2 and KC was decreased in the lungs of mice with *E. coli*‐induced sepsis receiving Tg MSCs compared with mice receiving PBS (Fig. 6B and C).

FIGURE 6
**Administration of dnHMGA1 Tg MSCs during *E. coli*‐induced sepsis decreased expression of IL‐6 and neutrophil chemokines in tissue**. C57BL/6 mice underwent sham or fibrin clot (*E. coli*) surgery. Six hours after surgery the mice received PBS, WT MSCs (2.5 × 10^5^), or dnHMA1 Tg MSCs (2.5 × 10^5^). At 24 h after surgery, lungs were harvested from each group and total RNA extracted. Quantitative real time PCR (qRT‐PCR) was assessed for the levels of (**A**) IL‐6, (**B**) MIP‐2, and (**C**) KC. qRT‐PCR of β‐actin was employed for normalization of each gene expression. Gene expression is presented as fold‐increase compared with sham. Data are represented as mean ± sem, a minimum of *n* = 8 in sham and *n* = 12 in sepsis groups. Analysis by one‐way ANOVA (*P *= 0.0011, *P *= 0.0028, and *P *= 0.0115, respectively), with significant comparison * versus sham, † versus PBS, and § versus WT MSCs using Kruskal–Wallis multiple comparisons test

**Figure:**
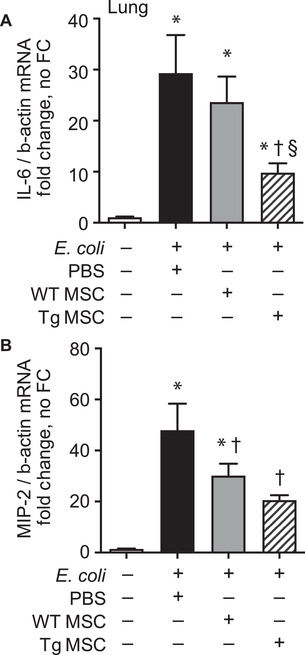

**Figure:**
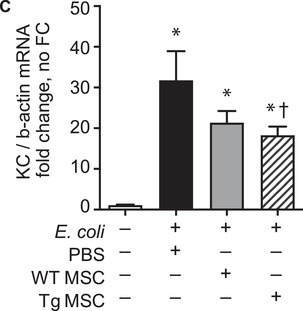

### 3.5 dnHMGA1 Tg MSCs increase the phagocytic function of neutrophils, and demonstrate increased production of SDF‐1

**FIGURE 7:**
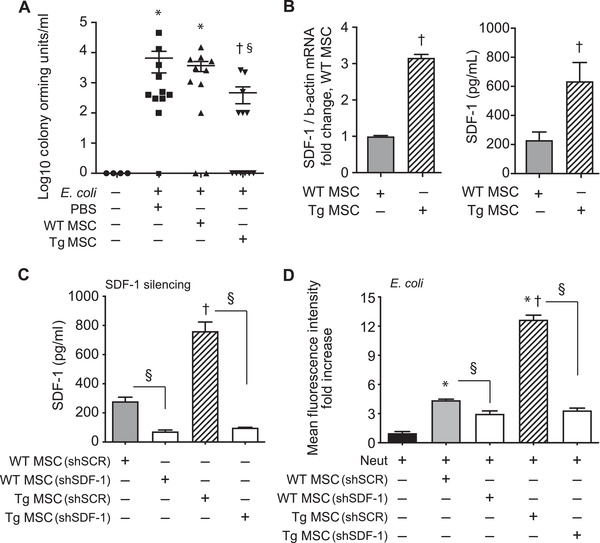
**Expression of dnHMGA1 transgene in MSCs improved their ability to interact with neutrophils and promote bacterial phagocytosis, in part through increased expression of SDF‐1**. (**A**) C57BL/6 mice underwent sham or fibrin clot (*E. coli*) surgery. Six hours after surgery the mice received PBS, WT MSCs (2.5 × 10^5^), or dnHMA1 Tg MSCs (2.5 × 10^5^). At 24 h after surgery, peritoneal lavage fluid was collected and analyzed for CFUs of bacteria (Log10). Data are presented as scatter dot plots (shown mean ± sem), *n* = 4 in sham and *n* = 11 in the fibrin clot groups receiving PBS, WT MSCs, or TG MSCs. Analysis by one‐way ANOVA (*P *= 0.0044), with significant comparison * versus sham, † versus PBS, and § versus WT MSCs using Kruskal–Wallis multiple comparisons test. (**B**) SDF‐1 mRNA (left panel) and protein (right panel) levels were assessed in WT and Tg MSCs. Quantitative real time PCR (qRT‐PCR) was used to assess SDF‐1 mRNA levels, whereas β‐actin was employed for normalization of gene expression. Gene expression is presented as fold‐increase compared with WT MSCs. Data are presented as mean ± sem, *n* = 3 per group. Analysis by unpaired *t*‐test (2 tailed), with significant comparisons † versus WT MSCs (*P *< 0.0001). SDF‐1 protein levels (pg/ml) were assessed by ELISA in conditioned medium of WT and Tg MSCs. Data are presented as mean ± sem, *n* = 4 per group. Analysis by unpaired *t*‐test (2 tailed), with significant comparisons † versus WT MSCs (*P *= 0.0278). (**C**) ELISAs for SDF‐1 were performed on cell culture supernatants of WT MSCs (shSCR and shSDF‐1) and Tg MSCs (shSCR and shSDF‐1). Data are presented as mean ± sem, *n* = 3 in shSCR and *n* = 4 in shSDF‐1 groups. Analysis was performed by one‐way ANOVA (*P* < 0.0001), with significant comparisons † versus WT MSC (shSCR), and § shSDF‐1 versus shSCR groups. (D) Isolated neutrophils were incubated with GFP‐labeled *E. coli* in the presence of WT or dnHMGA1 Tg MSCs, silenced for SDF‐1 (shSDF‐1) or scrambled control (shSCR). Data are presented as mean ± sem, *n* = 3 per group. Analysis by one‐way ANOVA (*P *< 0.0001), with significant comparisons * versus no MSCs, and † versus WT shSCR MSCs, and § shSDF‐1 versus shSCR MSCs

Eradication of the invading microorganism(s), along with subsequent resolution of the inflammatory response, leads to improved outcomes in sepsis.^20^, ^32^, ^33^ Assessment of peritoneal bacteria 24 h after fibrin clot surgery revealed decreased CFUs in mice receiving Tg MSCs, compared with mice receiving PBS or WT MSCs (Fig. 7A). Since neutrophils are a critical innate immune cell responsible for bacterial clearance, we next investigated the impact of the dnHMGA1 transgene on MSCs to promote neutrophil phagocytosis of *E. coli*. We recently demonstrated that SDF‐1 is an important paracrine factor produced by MSCs that improves neutrophil function during sepsis.^24^ Interestingly, the level of SDF‐1 mRNA in the cells, and SDF‐1 protein in the conditioned medium, was 3‐fold higher in Tg MSCs compared with WT MSCs (Fig. 7B). Thus, we next silenced SDF‐1 in Tg MSCs to determine whether the increased expression of SDF‐1 contributes to improved neutrophil phagocytosis, and thus bacterial clearance. In both WT and Tg MSCs, silencing of SDF‐1 (shSDF‐1) led to a marked decrease in protein expression compared with the scrambled control (shSCR; Fig. 7C). WT shSCR MSCs significantly increased the phagocytosis of *E. coli* by neutrophils, compared with neutrophils not exposed to cells (Fig. 7D). However, when neutrophils were exposed to Tg shSCR MSCs, the phagocytosis of *E. coli* by neutrophils was significantly enhanced compared with WT shSCR MSCs. Importantly, when SDF‐1 was silenced in Tg MSCs (Tg shSDF‐1 MSCs), the enhanced neutrophil phagocytosis was abolished, and decreased to the level of bacterial phagocytosis by WT shSDF‐1 MSCs (Fig. 7D).

## 4 DISCUSSION

HMGA proteins are nonhistone chromatin proteins that play a significant role in the biologic function of cells, especially those in a more primitive or undifferentiated state. For example, HMGA proteins are highly expressed during embryonic development, with the level of expression decreasing significantly in differentiated adult tissues.^10^ That being said, we have shown the mesenchymal cells from adult tissues, such as vascular smooth muscle cells, express low levels of HMGA1 and this expression can be induced by inflammatory or proliferative stimuli.^12^, ^13^ HMGA proteins are highly expressed in cancer cells, and have been used as tumor markers.^34^, ^35^ Expression of HMGA1 has also been noted in undifferentiated embryonic stem cells (ESCs) of both mouse and human origin, and the levels of expression decrease during cellular differentiation.^7^, ^36^ Thus, HMGA1 has an impact on cell growth, and overexpression can block differentiation or enhance self‐renewal of stem cells.^10^ In contrast to ESCs, much less is known about the biologic role of HMGA1 in MSCs. To explore this further, we investigated the impact of expressing dnHMGA1 in adipose‐derived MSCs.

MSCs were harvested from Tg mice overexpressing a dn form of HMGA1 (dnHMGA1) in mesenchymal cells that contains mutations in the second and third DNA‐binding domains,^18^ which prevents the dn construct from binding to DNA.^14^ Although we originally assessed the impact of dnHMGA1 on vascular smooth muscle cells, we now demonstrate that the dnHMGA1 Tg construct is also expressed in MSCs from the Tg mice, but not MSCs harvested from WT littermate mice. Expression of dnHMGA1 transgene did not change the expression of endogenous HMGA1, and there was no alteration in MSC phenotyping using flow cytometry. HMGA proteins have been suggested to play a role in adipogenesis^37^, ^38^; however, Tg MSCs harvested from adult mice showed no alteration of cellular differentiation into adipocytes, osteoblasts, or chondrocytes compared with WT MSCs. Although it is known that overexpression of HMGA1 is associated with cellular proliferation and neoplastic transformation,^39^ it has been suggested that exposure of MSCs to extracellular HMGA1 may actually reduce proliferation.^40^ Nevertheless, in the present study, dnHMGA1 Tg MSCs demonstrated less cell proliferation compared with WT MSCs, which is consistent with less growth potential in cells with disruption of the HMGA1 pathway.

It has previously been shown that elevated levels of HMGA1 were associated with increased production of cellular reactive oxygen species, and decreased efficiency of mitochondrial DNA repair due to oxidative injury.^41^ Interestingly, Tg MSCs were more resistant to death due to oxidative stress than WT MSCs and also produced less IL‐6 in the presence of PIS. Thus, we administered WT and Tg MSCs to WT mice after the onset of *E. coli*‐induced peritoneal sepsis. When cells were administered in 2 doses, Tg and WT MSCs had a very similar effect to improve survival of mice compared with fibroblasts or PBS controls. However, when the MSCs were injected in a single dose (50% fewer total cells), WT MSCs lost their efficacy, whereas the Tg MSCs remained beneficial with a significantly improved survival of septic mice compared with WT MSCs. It is feasible that less cell death of Tg MSCs in an environment of excess oxidative stress, and less production of proinflammatory mediators, may have contributed to their sustained efficacy. As shown previously, the dnHMGA1 construct expressed in the Tg mice cannot bind to DNA, but can interact with transcription factors (such as NF‐kB subunits).^14^ We believe that by sequestering transcription factors that regulate expression of proinflammatory mediators, dnHMGA1 influenced the response of the MSCs during sepsis, as evidenced by decreased IL‐6 production in dnHMGA1 MSCs after exposure to PIS compared with WT MSCs. This advantageous effect of Tg MSCs on mouse survival was associated with less cell death in organs inside (spleen) and outside (lung) of the peritoneum, compared with WT MSCs or no cell therapy.

To further evaluate additional consequences of experimental sepsis, we assessed the inflammatory response in the peritoneal fluid and tissues (lungs and spleens) of mice receiving Tg MSCs, WT MSCs, or no cell therapy. Previously we assessed dnHMGA1 Tg mice compared with WT mice after the onset of *E. coli*‐induced sepsis. Along with improved survival, the Tg mice demonstrated the ability of innate immune cells to reach the peritoneum, but decreased infiltration of inflammatory cells into tissue.^14^ In the present study, WT MSCs produced a decrease in the infiltration of neutrophils (Ly6G^+^ cells) compared with no cell administration, and injection of Tg MSCs led to an even further decrease in neutrophils in both the lungs and spleens of WT septic mice. However, MSCs (WT or Tg) did not limit infiltration of neutrophils into the peritoneal fluid. Thus, the MSCs limited nonspecific infiltration of neutrophils into tissues, but maintained neutrophil migration to the site of infection. Moreover, tissue production of IL‐6, and also the neutrophil chemokines MIP‐2 and KC were significantly decreased in mice receiving Tg MSCs. Although Tg MSCs expressed less IL‐6 when exposed to PIS, it is likely that a decrease in the inflammatory response in tissues (such as the lung) explains the decreased local production of IL‐6, which is associated with less tissue injury. To determine whether this effect on tissue injury and inflammation was also seen in another clinically relevant model of sepsis, we assessed lung tissue from mice after CLP surgery plus antibiotic therapy. MSCs were able to decrease cell death and neutrophil infiltration into the lungs, and Tg MSCs were able to promote a further decrease compared with mice receiving WT MSCs.

An important aspect of resolving the inflammatory response during sepsis is to eliminate the invading microorganism(s). In the mice receiving Tg MSCs, we found a decrease in the peritoneal bacterial counts compared with mice receiving WT MSCs or PBS at 24 h after the onset of *E. coli*‐induced sepsis. We recently demonstrated that expression of SDF‐1 in MSCs played a critical role in promoting neutrophil phagocytosis and bacterial clearance during sepsis.^24^ Moreover, HMGA1 is known to bind to the SDF‐1 promoter.^42^ Interestingly, we found that the expression of SDF‐1 was 3‐fold higher in Tg MSCs (mRNA), and equally increased in the conditioned medium (protein) of Tg MSCs, compared with WT MSCs. We previously demonstrated that WT MSCs increase neutrophil phagocytosis, in part via paracrine actions.^17^, ^20^, ^24^ This improved neutrophil response in the presence of WT MSCs (shSCR) was also apparent in the present study; however, Tg MSCs (shSCR) significantly enhanced the increase in bacterial phagocytosis by neutrophils. Moreover, silencing of SDF‐1 (shSDF‐1) in Tg MSCs eliminated the enhanced phagocytosis by neutrophils. These data confirm that increased levels of SDF‐1 in dnHMGA1 Tg MSCs were critical for the improved phagocytic response by neutrophils, and thus bacterial clearance.

Previously, we reported the impact of expressing a dnHMGA1 transgene to improve outcomes in endotoxin exposure and bacterial sepsis, with a focus on vascular smooth muscle cells.^14^ In the present study, we advanced our understanding of the role of HMGA1 in another mesenchymal cell, MSCs from Tg mice. Taking a cell therapeutic approach, we demonstrate that Tg MSCs have functional benefits beyond WT MSCs during experimental sepsis. These favorable results include sustaining the efficacy of Tg MSCs when administering fewer cells in a single dose compared with WT MSCs. We propose that better clearance of bacteria from the peritoneum of WT mice receiving Tg MSCs, through enhanced neutrophil phagocytosis, played a critical role in the improved outcome, allowing resolution of the inflammatory response, less tissue injury, and ultimately increased survival. Thus, disrupting the HMGA1 pathway in MSCs provides an improved therapeutic response during bacterial sepsis.

## AUTHORSHIP

M‐YK, SG, APC, JAL, SEC, SWC, XL, and MAP helped to design the research studies. M‐YK, SG, JN, APC, JH, and BI conducted the experiments and acquired the data. M‐YK, SG, APC, and MAP analyzed the data. M‐YK, SG, and MAP wrote and edited the manuscript. M.‐Y.K. and S.G. contributed equally to this work.

## DISCLOSURES

The authors declare no conflicts of interest.

## Supporting information

Supporting Table S1
