# Supplementary material for: Mesenchymal stromal cells expressing a dominant‐negative high mobility group A1 transgene exhibit improved function during sepsis
Source: J Leukoc Biol. 2021 Jan 13;110(4):711–22. doi: 10.1002/JLB.4A0720-424R (PMC8275698; doi:10.1002/JLB.4A0720-424R)
Supplement: Supplementary file 1 — Supporting Table S1 [file JLB-110-711-s001.pdf]

Supplemental Table 1. Antibodies used for flow cytometry cell phenotyping

Antibody	Company	Catalog number
CD105	Biolegend	120413
CD73	Biolegend	127210
CD90.2	eBioscience	12-0903-81
CD29	eBioscience	17-0291-80
CD44	eBioscience	12-0441-82
Sca-1	eBioscience	12-5981-82
Stro1	Invitrogen	39-8401
c-kit (CD117)	eBioscience	25-1171
CD45	Biolegend	103108
MHCII (I-A/I-E)	eBioscience	17-5321-82
